# Supplementary material for: A mechanism for reconciling the synchronisation of Heinrich events and Dansgaard-Oeschger cycles
Source: Nat Commun. 2024 Apr 5;15:2961. doi: 10.1038/s41467-024-47141-7 (PMC10997585; doi:10.1038/s41467-024-47141-7)
Supplement: Supplementary file 1 — Supplementary Information [file 41467_2024_47141_MOESM1_ESM.pdf]

Supplementary information for the manuscript "A mechanism for reconciling the synchronisation of Heinrich events and Dansgaard-Oeschger cycles"

Clemens Schannwell[1*], Uwe Mikolajewicz[1], Marie-Luise Kapsch[1] & Florian Ziemen[2]

[1]*Max Planck Institute for Meteorology, Bundesstraße 53, 20146 Hamburg, Germany*

[2]*Deutsches Klimarechenzentrum, Bundesstr. 45a, 20146 Hamburg, Germany*

Correspondence Correspondence and requests for materials should be addressed to Clemens Schannwell (email: clemens.schannwell@mpimet.mpg.de).

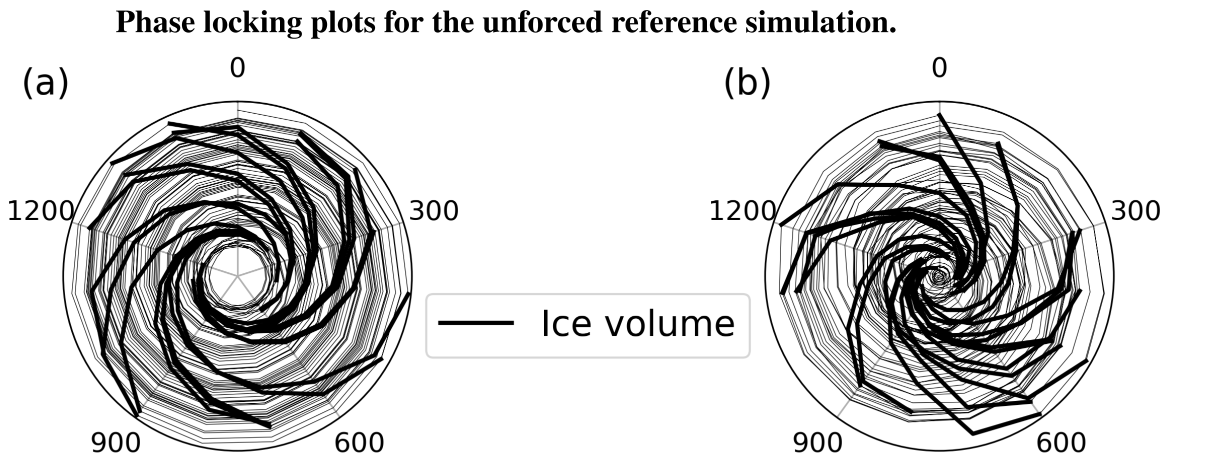

Supplementary Figure 1. Similar to Fig. 2, but for the reference simulation. (a) shows Hudson ice stream and (b) Mackenzie ice stream. Radial ice volume axes range from $4\times10^{15} - 5.9\times10^{15}\mathrm{m}^3$ and $3.85\times10^{15} - 4.95\times10^{15}\mathrm{m}^3$ for Hudson ice stream and Mackenzie ice stream, covering $\sim$4.8 and $\sim$2.8 m of sea-level equivalent, respectively.

Phase locking plots for complete ensemble of the prescribed Dansgaard-Oeschger cycle.

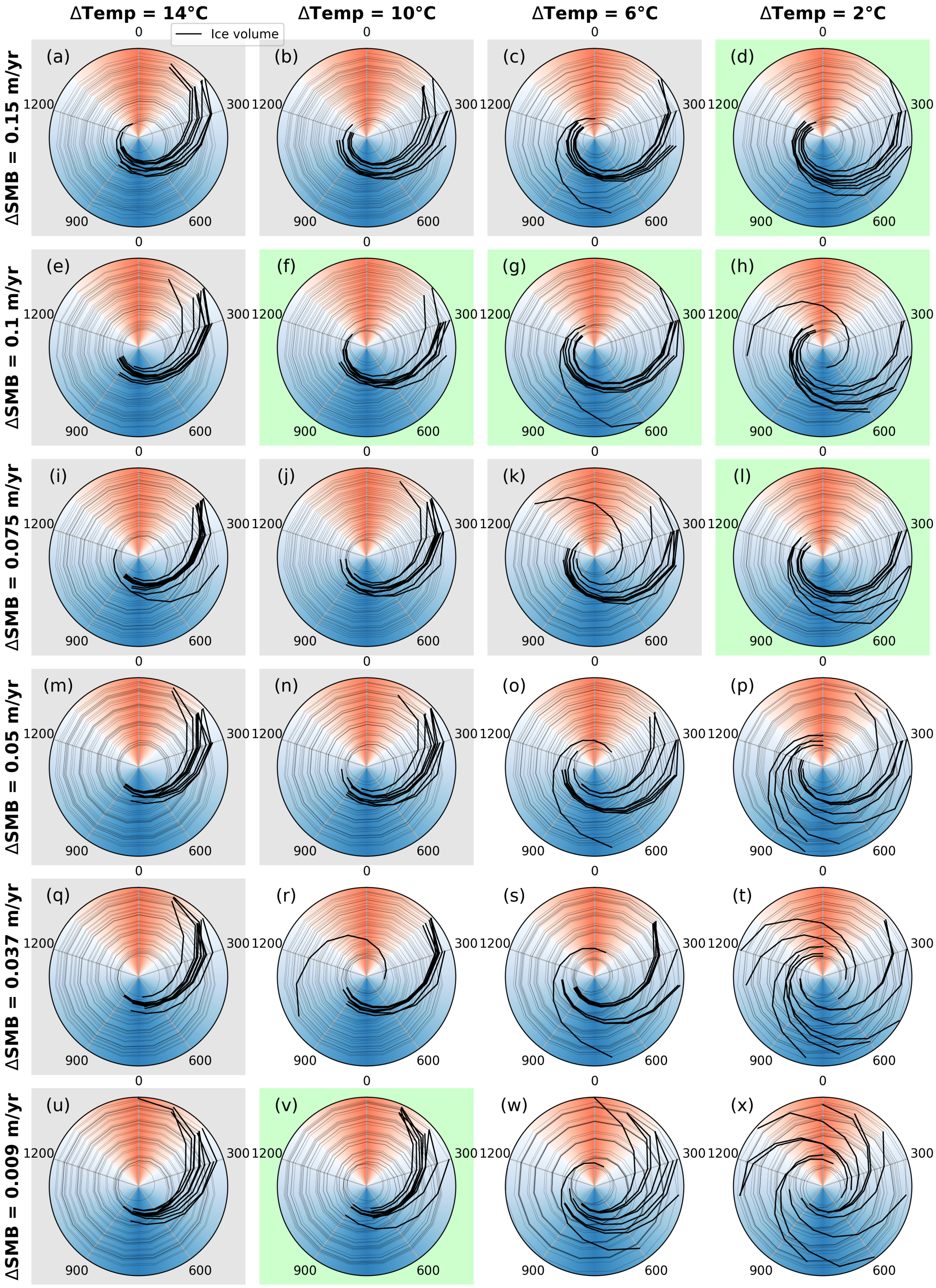

Supplementary Figure 2. Similar to Fig. 2, but for the complete ensemble of prescribed Dansgaard-Oeschger cycle forcings.

Phase locking plots for a single atmospheric forcing.

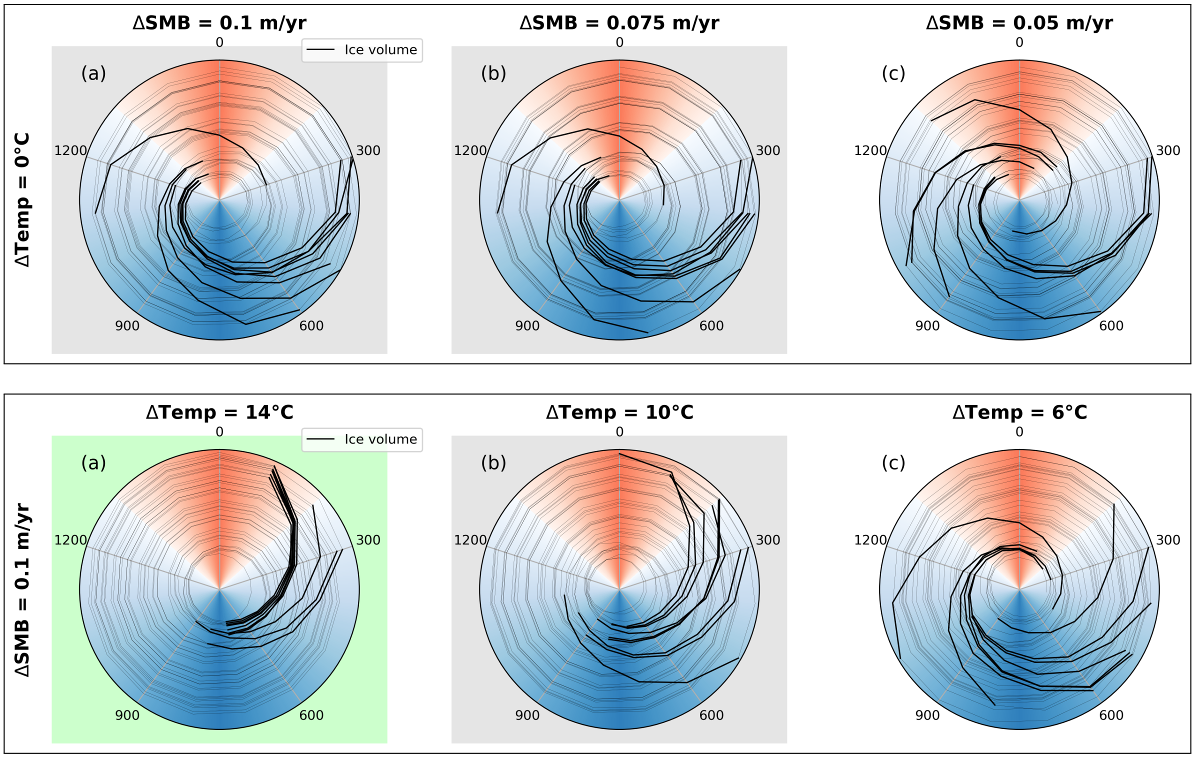

Supplementary Figure 3. Similar to Fig. 2, but for surface mass balance only (upper panel) and surface temperature only (lower panel) perturbations. Radial ice volume axes range from $4\times10^{15} - 5.9\times10^{15}\mathrm{m}^3$, covering $\sim$4.8 meters of sea-level equivalent..

Phase locking plots for different Dansgaard-Oeschger cycle lengths.

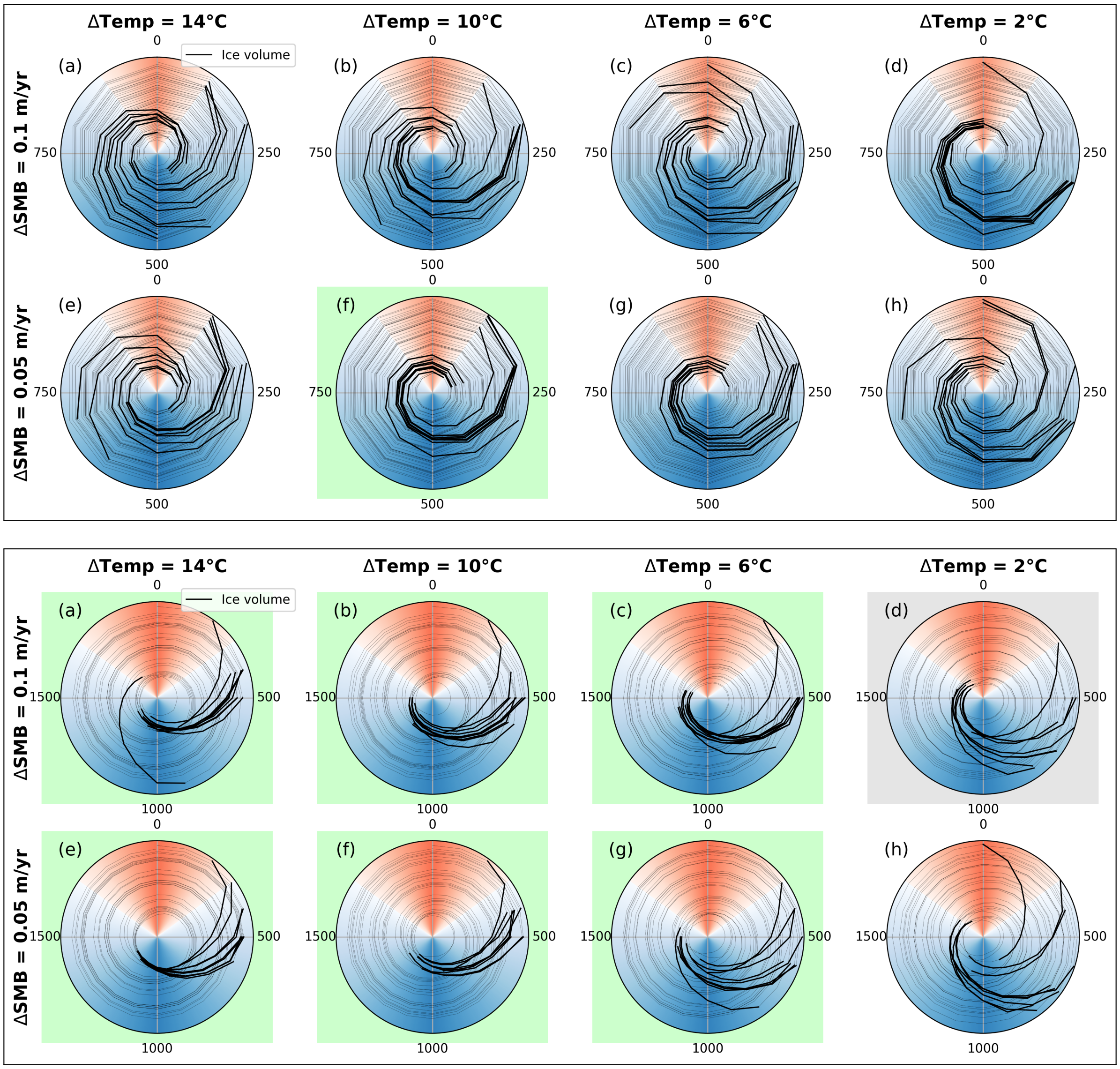

Supplementary Figure 4. Similar to Fig. 2, but from the ensemble of 1,000 year-long (upper two panels) and 2,000 year-long (lower two panels) prescribed Dansgaard-Oeschger cycle forcings. Radial ice volume axes range from $4\times10^{15} - 5.9\times10^{15}\text{m}^3$, covering ∼4.8 meters of sea-level equivalent..

Phase locking plots for randomly varying Dansgaard-Oeschger cycle lengths.

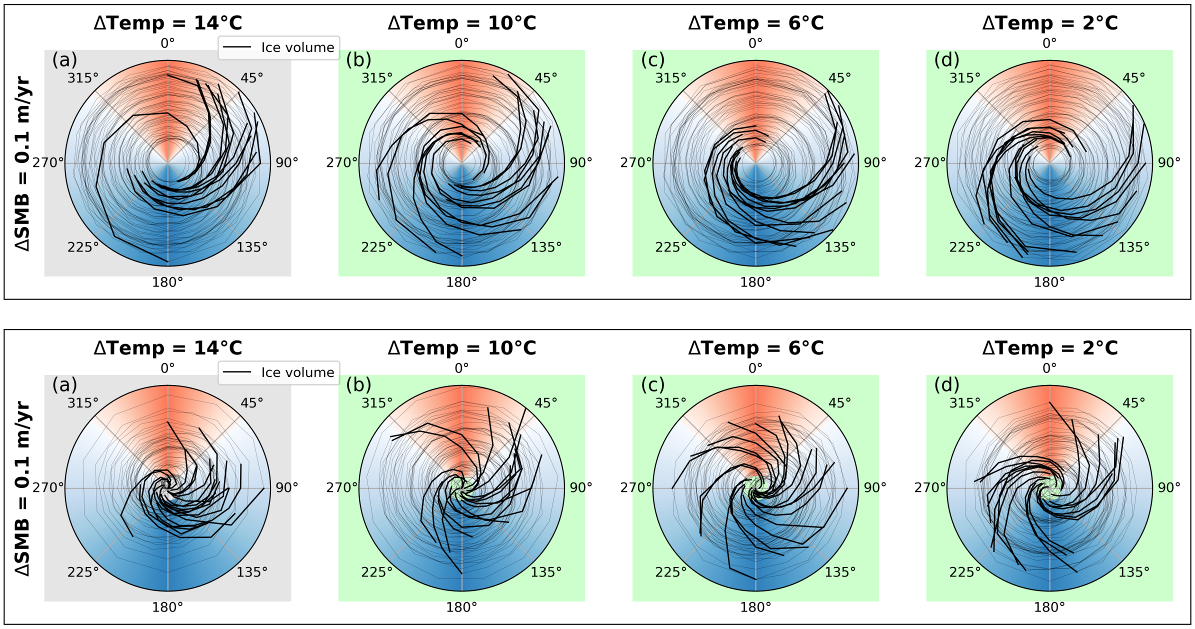

Supplementary Figure 5. Similar to Fig. 2, but for a stochastically varying Dansgaard-Oeschger cycle length between 1,000 and 2,000 years. Note that the circle's circumference here has units of degrees (°) to allow the plotting of all events in one Figure. Upper panel shows the Hudson ice stream. Lower panel shows the Mackenzie ice stream.

Atmospheric forcing for the spatially-varying Dansgaard-Oeschger cycle.

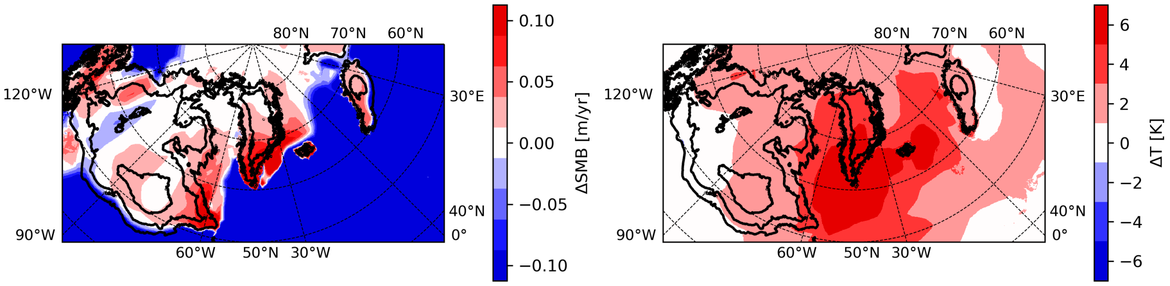

Supplementary Figure 6. Spatially-varying Dansgaard-Oeschger cycle forcing as derived from the fully coupled MIS3 simulation with interactive ice sheet and solid earth components. Left panel shows surface mass balance. Right panel shows surface temperature. Black contours depict 1000 m ice thickness contours.

Phase locking plots for spatially-varying Dansgaard-Oeschger cycle lengths.

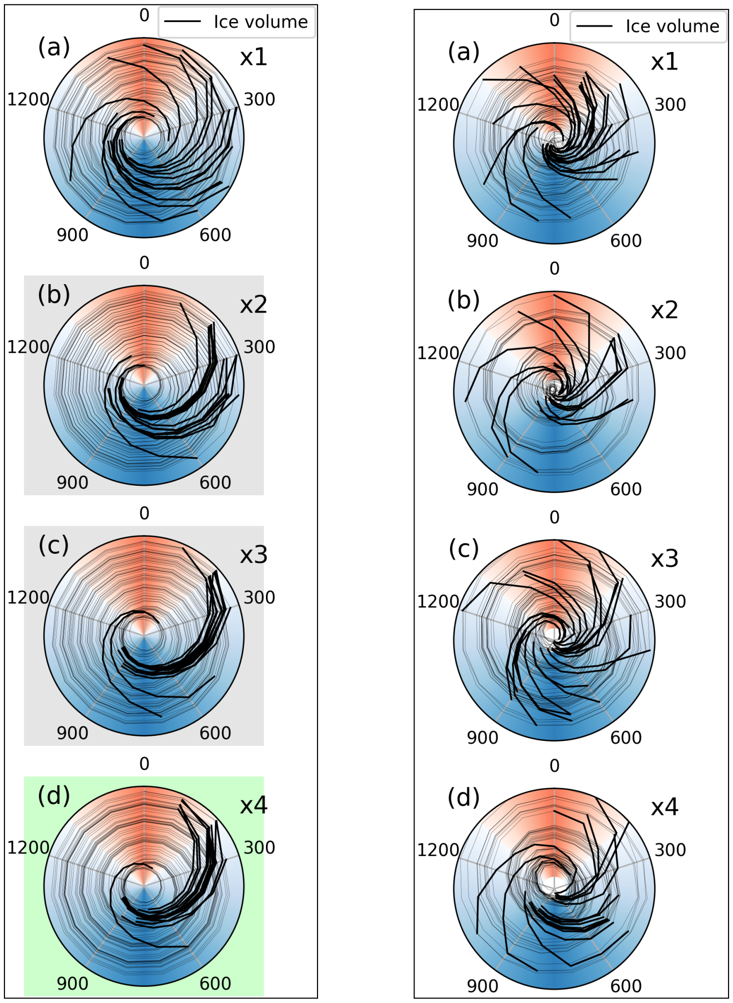

Supplementary Figure 7. Similar to Fig. 2, but from the ensemble of spatially-varying prescribed Dansgaard-Oeschger cycle forcings. Left panel shows the Hudson ice stream. Right panel shows the Mackenzie ice stream. Radial ice volume axes range from $4\times10^{15} - 5.9\times10^{15}\mathrm{m}^3$ and $3.85\times10^{15} - 4.95\times10^{15}\mathrm{m}^3$ for Hudson ice stream and Mackenzie ice stream, covering $\sim$4.8 and $\sim$2.8 m of sea-level equivalent, respectively.

Phase locking plots for complete ensemble of the prescribed Dansgaard-Oeschger cycle.

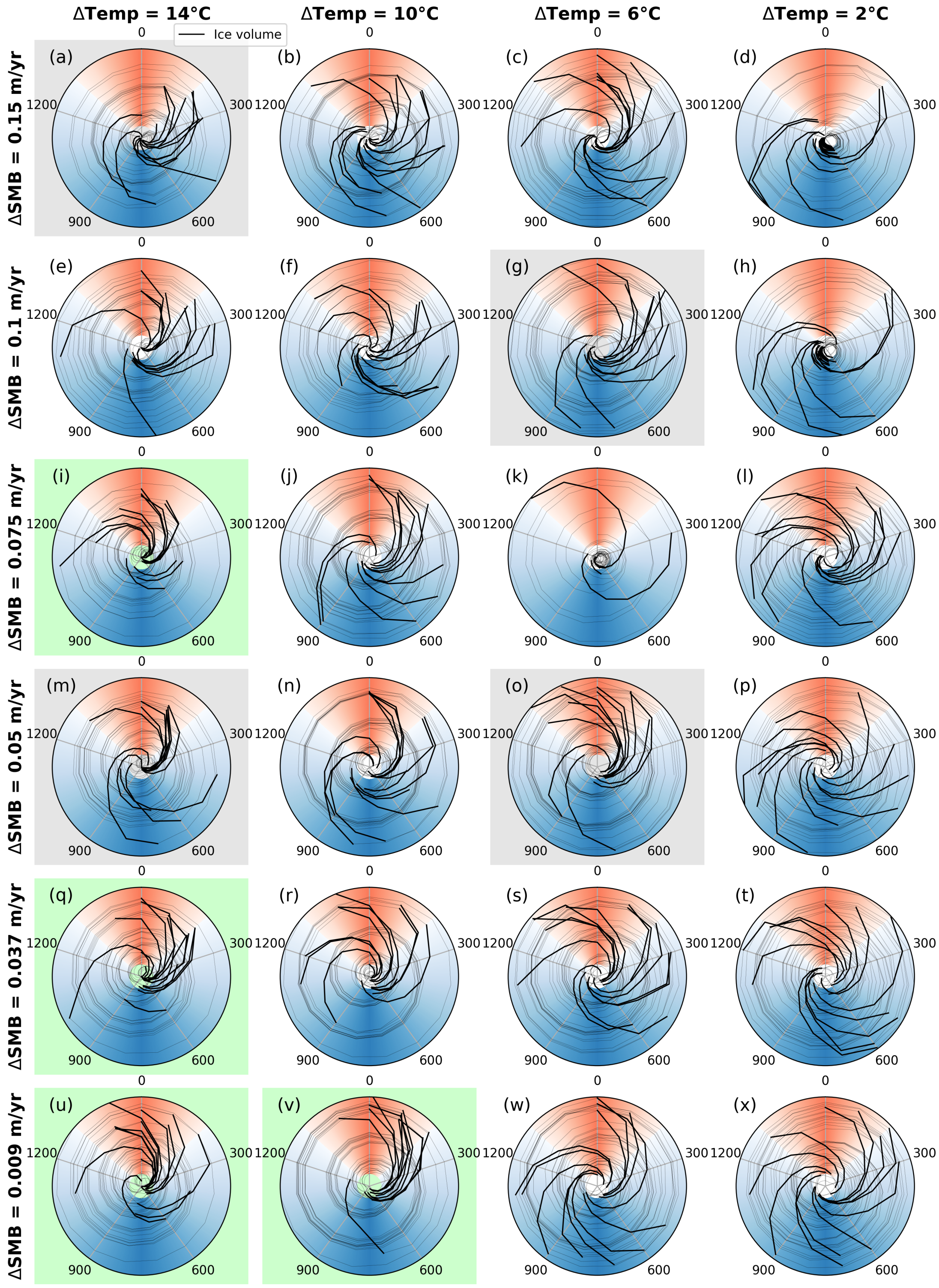

Supplementary Figure 8. Similar to Supplementary Fig. 2, but for the Mackenzie ice stream. Radial ice volume axes range from $3.85\times10^{15} - 4.95\times10^{15}\text{m}^3$, covering $\sim$2.8 meters of sea-level equivalent.

Phase locking plots for different Dansgaard-Oeschger cycle lengths.

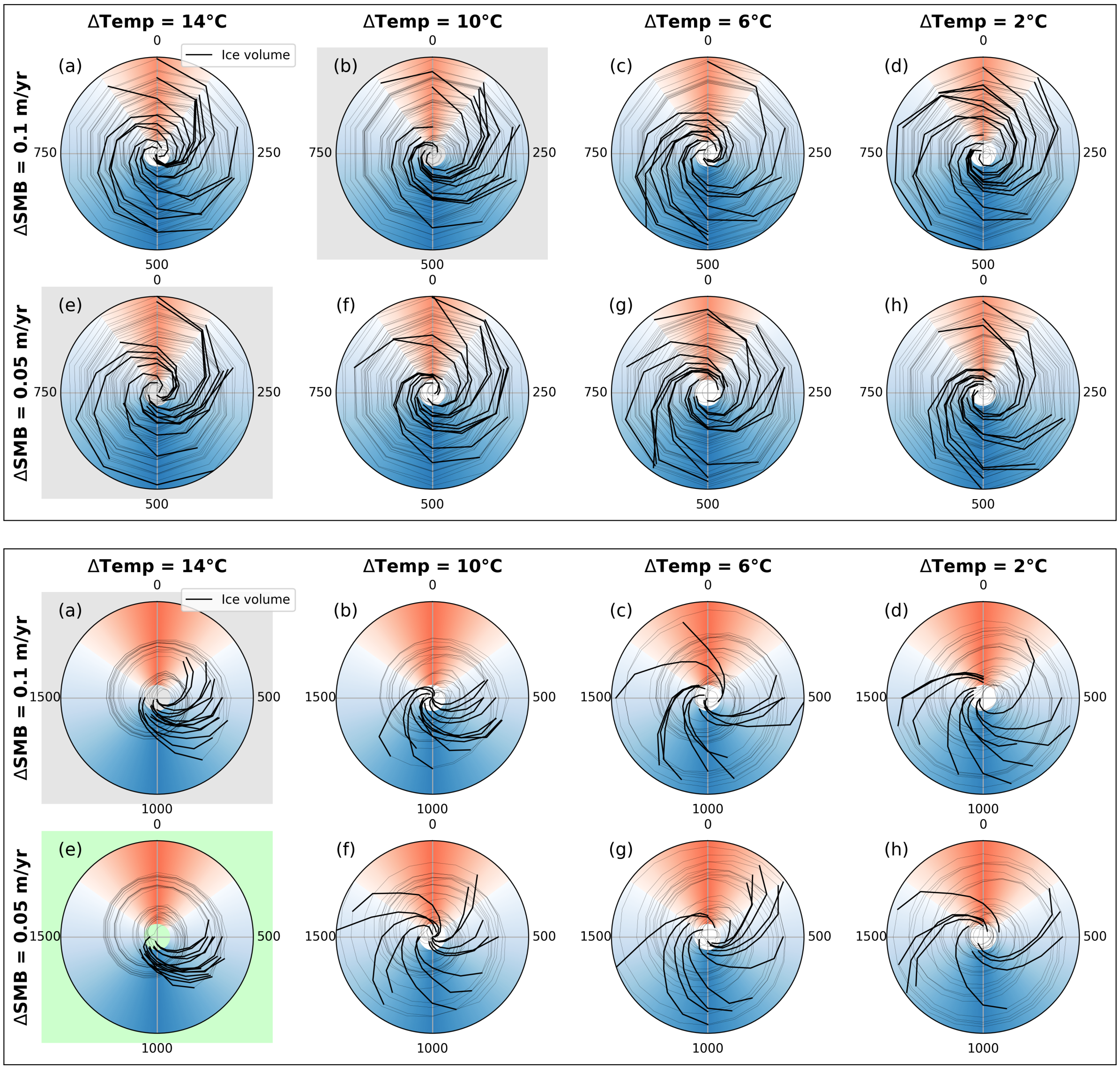

Supplementary Figure 9. Similar to Fig. 2, but for the Mackenzie ice stream and from the ensemble of 1,000 year-long (upper two panels) and 2,000 year-long (lower two panels) prescribed Dansgaard-Oeschger cycle forcings. Radial ice volume axes range from $3.85\times10^{15} - 4.95\times10^{15}\text{m}^3$, covering $\sim$2.8 meters of sea-level equivalent.
